# Proximal and contextual correlates of childhood stunting in India: A geo-spatial analysis

**DOI:** 10.1371/journal.pone.0237661

**Published:** 2020-08-20

**Authors:** Ashish Kumar Gupta, K. G. Santhya

**Affiliations:** Population Council, New Delhi, India; University of Botswana, BOTSWANA

## Abstract

**Background:**

Globally, India is home to every third child affected by stunting. While numerous studies have examined the correlates of childhood stunting (CS) in India, most of these studies have focused on examining the role of proximal factors, and the role of contextual factors is much less studied. This study presents a comprehensive picture of both proximal and contextual determinants of CS in India, expanding the current evidence base. The present study is guided by the WHO conceptual framework, which outlines the context, causes, and consequences of CS.

**Data and methods:**

The study used exploratory spatial data analysis tools to analyse the spatial pattern and correlates of CS, using data from the fourth round (2015–16) of the National Family Health Survey (NFHS-4) and the 2011 Census of India.

**Results:**

The study findings reiterate that CS continues to be high in India, with several hot spot states and districts, and that children from the central and eastern region of the nation, namely, Bihar, Jharkhand, Madhya Pradesh, and Uttar Pradesh are particularly vulnerable. Our analysis has identified six risk factors—maternal short stature, large household size, closely spaced births, prevalence of hypertension among women, household poverty, open defecation, and extreme temperature—and four protective factors—female education, access to improved drinking water, dietary diversity among children, and iron and folic acid (IFA) supplementation during pregnancy.

**Conclusions:**

The study highlights the need for investing in pre-conception care, addressing both demand- and supply-side barriers to increase the coverage of nutrition-specific interventions, implementing programmes to promote the intake of healthy foods from an early age, providing contraceptive counselling and services to unmarried and married adolescents and young women and men, and universalizing quality primary and secondary education that is inclusive and equitable to avert the burden of childhood stunting in India.

## Background

India, with 47 million stunted children (0–5 years), has the highest number of stunted children worldwide and is home to every third stunted child globally [1]. The recent trends show that the prevalence of CS has declined by 10 percentage points from 48 percent in 2005–06 to 38 percent in 2015–16 [2,3]. Even so, with the current pace of decline of one percent annually, India is unlikely to achieve the target set by the National Nutrition Mission (NNM) to reduce CS prevalence to 25 percent by 2022 [4] nor the global nutrition target and the Sustainable Development Goals target to reduce the proportion of stunted children under five by 40 percent by 2025 [5,6].

The UNICEF’s framework on the causes of malnutrition among children [7] and its various adaptations, for example, by the WHO [8,9] and the Lancet Maternal and Child Nutrition Series [10,11], delineated a wide range of risk and protective factors underlying CS—contextual (community and societal factors) and proximal factors (household and family factors, breastfeeding and complementary feeding practices and infections). Informed by these frameworks, numerous studies have examined the correlates of CS in India; indeed, a search on PubMed and Google Scholar has identified at least 18 peer-reviewed journal articles published in the last one decade or so on the correlates of CS in India. A review of these studies shows that their focus has been by and large skewed towards examining the role of proximal factors. Specifically, several studies have observed a direct association between CS and maternal factors such as poor maternal nutrition, short maternal stature, low levels of maternal education, and early age at marriage and childbirth [12–20]. These studies have also shown that CS in India is significantly correlated with household-level poverty and other forms of deprivations such as food shortage, lack of access to water and sanitation facilities, and limited access to clean cooking fuel [15,17,21,22,45]. In comparison, fewer studies have explored the relationship between CS and breastfeeding and complementary feeding practices and infections; the few that are available have noted the importance of infection control, dietary diversity, exclusive breastfeeding, and the uptake of micronutrient supplements in reducing CS in India [12,14,15,18,20]. As in the global literature [8], the role of contextual factors is much less studied than that of proximal factors of CS in India. We came across just four articles that assessed the role of contextual factors such as climatic conditions, food production, access to healthcare facilities, and macroeconomic development [21–24]. These studies have by and large explored the role of selected contextual factors in isolation; moreover, the analysis presented in some of these studies was limited to small geographies. In short, studies that assessed the role of proximal and contextual factors comprehensively are few and far between in India.

One of the salient features of CS in India is the substantial heterogeneity in its prevalence across states and districts [13,18]. The 2015–16 National Family Health Survey (the Indian equivalent of Demographic Health Survey) shows that CS prevalence ranged from 19 percent in Kerala to 48 percent in Bihar and district-level prevalence ranged from 12 percent in Ernakulam (Kerala) to 65 percent in Bahraich (Uttar Pradesh) [2]. These variations point towards differential contextual and socioeconomic risk factors underlying CS in India, and these factors may operate differently across geographic spaces [18]. Although epidemiologists and medical geographers have long acknowledged geographic space as an important determinant of health outcome disparities [25–31], studies that have explored spatial heterogeneity in CS, particularly district-level heterogeneity in CS, are limited in India. The majority of the available studies on the correlates of CS used individual or state as the unit of analysis, mainly because of the lack of district-level data on CS until recently. A few recent studies, taking advantage of the district-level data from the most recent NFHS conducted in 2015–16, have explored the district-level heterogeneity in CS and its correlates [17,18,22]. Although these studies offer rich insights on the district-level heterogeneity in CS in India, methodological limitations remain. None of these studies, for example, has drawn on the rich unit-level data from the NFHS-4 that could have enabled a comprehensive analysis of both proximal and contextual determinants of CS in India.

Triangulating data from the 2015–16 NFHS and the 2011 Indian census, this paper examines the proximal and contextual determinants of CS in India.

## Material and methods

### Data and sample details

Data for the present study were drawn primarily from the 2015–16 NFHS [2]. The 2015–16 round of the survey was designed to provide representative data on a range of population, health, and nutrition indicators at the district, state, and national levels. The NFHS-4 applied multistage systematic clustered sampling design, and data were collected from 601,509 households with a response rate of 98 percent—that is, from 699,686 women aged 15–49 with a response rate of 97 percent and 112,122 men aged 15–54 with a response rate of 92 percent. Data were also available for 259,627 children aged 0–5. In addition to face-to-face interviews with adult women and men, the NFHS-4 collected biomarker information, including measurements of height, weight, and haemoglobin for children (more details about NFHS-4 are available at [2]). We also drew on data pertaining to population density, urbanization, access to electricity, and extreme temperature from the 2011 Census of India, and these data were aggregated at the district level and merged with district-level variables drawn from the NFHS-4.

The unit of analysis in the paper is the district. We included districts for which data points for the outcome variable and all the explanatory variables used in our analysis were available and excluded those districts for which data points were missing for any one of these variables. As such, the analytical sample included 640 districts for the descriptive analysis and 611 districts for regression-based analysis, and the remaining 29 districts were excluded from the regression-based analysis because of missing observations for selected explanatory variables such as dietary diversity, consumption of 100 or more IFA tablets, food supplementation through Integrated Child Development Services (ICDS), and maximum daytime temperature. We compared the outcome measure and selected characteristics of districts included in the analytical sample for the regression-based analysis (N = 629) and for those that were excluded (N = 11), and we found that the prevalence of CS or such characteristics as female education, access to electricity, or dietary diversity did not differ between the included and the excluded districts (see S1 Table). However, the excluded districts were poorer, less urban, and had a higher concentration of disadvantaged castes compared with districts included in the analysis. We note that the spatial regression analysis model included poverty levels and the proportion of disadvantaged groups at the district level.

#### Outcome variable

The outcome variable is the district-level prevalence of CS. The height measurement was available for children aged 0–59 months, which allowed estimating the proportion of children who were stunted or chronically undernourished. The height of children aged 24–59 months was measured with the Seca 213 stadiometer, and the recumbent length of children under two years or those less than 85 centimeters was measured using the Seca 417 infantometer. Children whose height-for-age Z-score was below minus two standard deviations (-2 SD) from the median of the reference population were considered short for their age (stunted).

#### Explanatory variables

The selection of explanatory variables, both proximal and contextual factors, is guided by the WHO conceptual framework, which outlines the context, causes, and consequences of CS [8]. We grouped them under three broad categories: (1) maternal and home environment factors (maternal stature, maternal anaemia, short birth interval, self-reported prevalence of elevated blood pressure among women, household size, female education, household poverty measured by household wealth index [excluding access to drinking water and access to toilet facilities as these variables were included in the model separately], household access to improved sources of drinking water, and household practice of open defecation); (2) breastfeeding and complementary feeding practices and infections among young children (early initiation of breastfeeding, dietary diversity, and prevalence of diarrhoea); and (3) contextual factors (awareness of nutrition-promoting actions, reach and uptake of nutrition-specific interventions, such as iron and folic acid supplementation during pregnancy, uptake of micronutrient supplement among young children, food supplementation for pregnant and lactating women, household access to health insurance schemes, population density, urbanization, village electrification, and climatic conditions). Table 1 provides a brief description and the district-level summary statistics of the outcome and explanatory variables used in the analysis. We note the results were based on the aggregated district-level data, and therefore, the national averages for the outcome and explanatory variables presented in this paper may not necessarily match exactly with the national averages obtained based on unit-level data.

**Table 1:** Description and district-level summary statistics of outcome and explanatory variables used in the analysis.

Variables	Definition	Mean	Standard deviation	Min.	Max.
**Dependent variable**					
**Childhood stunting prevalence**	% children aged (0–5) with height-for-age Z-score below minus two standard deviations from the median of the reference population^1^	35.8	9.9	12.2	64.9
**Independent variables—Maternal and home environment factors**					
**Maternal stature**	% of women aged 15–49 who gave birth in the 5 years preceding the survey, with less than 145 cm height^1^	10.4	5.8	1.3	29.4
**Maternal anaemia**	% of pregnant women aged 15–49 years who were moderately or severely anaemic^1^	25	11.7	0	67.9
**Short birth interval**	% of non-first birth within 24 months of the preceding birth in preceding 5 years^1^	25	7.9	0	44.6
**Self-reported prevalence of elevated blood pressure among women aged 15–49**	% of women aged 15–49 who gave birth in the last five years who reported that they were told by a doctor or health professional two or more occasions that they have hypertension or high blood pressure	8.9	7.0	0.94	63.2
**Household size**	% of households with more than six members^1^	34	12.8	6.7	73.1
**Female education**	% of women aged 15–49 who had completed 10 or more years of schooling^1^	34.6	14.5	9	86.3
**Household poverty**	% of households belonging to the poorest quintile of the household wealth index^1^,^3^	23.8	12.8	0.29	75.1
**Access to improved drinking water sources**	% of households with access to drinking water from piped water, public taps, standpipes, tube wells, boreholes, protected dug wells and springs, rainwater, and community reverse osmosis (RO) plants^1^	88.1	12.5	32.2	100
**Open defecation**	% of households practising open defecation^1^	38.5	27.7	0	89.9
**Independent variables—Complementary and breastfeeding practices and infections**					
**Early initiation of breastfeeding**	% of women aged 15–49 who started breastfeeding their newborn within the first hour of birth^1^	45.1	16.5	13.3	89.0
**Dietary diversity**	Ratio of children who received minimum dietary diversity, i.e., consumed items from more than four food groups during the previous day to the total number of children (aged 6–23 months)^1^	23.9	14.9	1.1	79.9
**Prevalence of diarrhoea**	Prevalence of diarrhoea (reported) among children under five years of age in the 2 weeks preceding the survey^1^	8.4	5.1	0.0	44.8
**Independent variables–Contextual factors**					
**Awareness of nutrition-promoting actions**	% of women who were aware of oral rehydration salt (as a proxy for health- and nutrition-awareness promotion activities)^1^	83.8	11.7	39.0	99.0
**Consumption of 100 or more IFA tablets**	% of women who gave birth in the five years preceding the survey and who consumed 100 or more IFA tablets during the antenatal period^1^	30.9	19.2	0.0	89.0
**Micronutrient intake**	% of children aged 9–59 months who received Vitamin A supplement in the 6 months preceding the survey^1^	59.7	17.3	12.8	94.5
**Food supplementation through Integrated Child Development Services (ICDS)**	% of children under six years whose mother received food supplements from the ICDS programme during pregnancy or lactation^1^	49.4	23.4	0.0	96.0
**Access to health insurance schemes**	% of households covered by any health insurance scheme^1^	25.8	22.1	0.6	86.0
**Population density**	Persons per square kilometer^2^	936.2	3053.3	0.9	36155.0
**Urbanization**	% of population residing in urban areas^2^	26.1	21.4	0.0	100.0
**Access to electricity**	% of villages electrified^2^	65.5	28.3	1.0	99.0
**Climatic conditions**	Extreme temperature in Celsius^2^	39.8	6.3	0.0	56.3
**Social Caste**	% of SC/ST population	36.8	22.7	0.5	99.9
**Religion**	% of Hindu population	75.1	27.5	0	99.8

Data sources: ^1^ National Family Health Survey 2015–16

^2^ Indian Census 2011

^3^; Wealth score aggregate after excluding access to drinking water and access to toilet facility.

#### India digital map

The district-level shape file (digital map) of India was obtained from GitHub at https://github.com/datameet/maps/tree/master/Districts. The digital map has been used under the Creative Commons Attributions 2.5 India license. The shape file was created using the administrative atlas of Census 2011, India. And the map was projected in WGS 1984 UTM zone 43N.

### Analytical approach

We first computed global Moran’s I, a commonly used indicator of spatial autocorrelation. The Moran’s I values range from −1 to 1. The value “1” means perfect positive spatial autocorrelation when similar values cluster together, while “−1” suggests perfect negative spatial autocorrelation when dissimilar values cluster together [32]. We used GeoDa 1.6.0 software to compute Moran’s I statistic and for further spatial analysis [32]. We used rook’s contiguity weight for estimating all the geo-spatial statistics and geo-spatial regressions [32]. We calculated two spatial weight matrix—one for all 640 districts for the univariate and bivariate spatial analysis and one for 611 districts for the regression analysis.

We generated local indicators of spatial association (LISA) map to detect spatial clusters with high value with similar neighbours (High-High) or hot spots and location with low value with similar neighbours (Low-Low) or cold spots. We also constructed quintile maps for the explanatory variables, drawing on the district-level prevalence value for each of the explanatory variables to capture spatial heterogeneity in the potential risk and protective factors. We estimated bivariate Moran’s I to explore the spatial association between CS and each of the explanatory variables. This indicates the magnitude and strength of correlation between outcome indicator (CS) at a given district and the average value of the explanatory variable in neighbouring districts.

The Moran’s I statistics value shows strong spatial clustering in CS across the districts of India (Global Moran’s I of 0.653, p<0.001). The presence of spatial autocorrelation in stunting across the districts can lead to correlation among error terms, rendering the ordinary least square (OLS) estimator inappropriate owing to violation of its underlying assumption of independence of error terms. Therefore, we used spatial regression technique to examine the determinants of CS. We ran simple as well as robust Lagrange Multiplier (LM) (lag) and Lagrange Multiplier (error) tests to decide the type of spatial dependence in the data. The LM test results are presented in Table 2. The simple LM test showed that both spatial lag model (SLM) and spatial error model (SEM) can be fitted to model the risk factors of CS (LM significant at 5% level). However, the robust LM test showed that SLM was not significant at five percent level of significance, while SEM was significant even at one percent level. Therefore, we decided to fit SEM to examine the association between CS and potential protective and risk factors. We also decided to fit SEM, because there could be explanatory variables that could not be included in the model owing to non-availability of the data at district level that may have an effect on the prevalence of childhood stunting.

**Table 2:** Results of diagnostic test of spatial dependence in OLS and other spatial models.

Test	MI/DF	value	Probability
**Moran’s I**	0.3169	13.4	0.000
**LM (Lag)**	1.00	93.28	0.000
**Robust LM (Lag)**	1.00	3.4	0.063
**LM (error)**	1.00	161.24	0.000
**Robust LM (error)**	1.00	71.3899	0.000

The SEM can be represented as:

Y=βX+ε

ε=λWϵ+μ

where, λ is spatial autoregressive parameter and the errors μ are independently and identically distributed. Thus, this is a special case of regression with a non-spherical error term. The spatial regression takes into account proximity among geographical units through the weight matrix W.

We tested the normality of residuals of prevalence of CS, using a normal probability plot (P-P plot), and found that the distribution was not overtly skewed. The plot of observed percentage of CS against the fitted percentage of CS confirms the assumption of conformity with normality. Collinearity between variables was also tested, using variance inflation factors (VIFs), which measure the strength of pairwise correlations between variables. In the full model, urbanization variable was found to be highly correlated with several variables, which resulted in a mean VIF of more than 10. Therefore, we excluded urbanization from the final full model and the mean VIF value for the final model was 2.2, which indicated a low level of multicollinearity (see S2 Table). The VIF value was lower than 4.0 and tolerance was more than 0.2 for all the variables included in the final model, which are considered acceptable cut-offs for VIF and tolerance in the available literature [33].

We fitted four SEM models. We included maternal and home environment variables in Model 1, indicators measuring infant and young children feeding practices and prevalence of infections in Model 2, and contextual-level variables in Model 3. The final model explored the relationship among all three sets of explanatory variables.

### Ethics statement

The respondents in the NFHS undergo an informed consent process for participation in the survey after approval of the protocol by the institutional review board of the IIPS. These NFHS datasets are available for download from the DHS program after registration through the weblink https://dhsprogram.com/what-we-do/survey/survey-display-355.cfm. This study was a secondary data analysis of de-identified data; therefore, ethics committee approval was not obtained.

## Results

### Spatial variation in childhood stunting

Findings presented in Table 1 show that over one-third of children were stunted in India. There were 231 districts in which the stunting prevalence was very high, that is, more than 40 percent of children were stunted. More than 80 percent of these districts were situated in the states of Bihar, Jharkhand, Madhya Pradesh, and Uttar Pradesh. In fact, Bihar and Uttar Pradesh together accounted for one-third of all stunted children in India.

**Fig 1:**
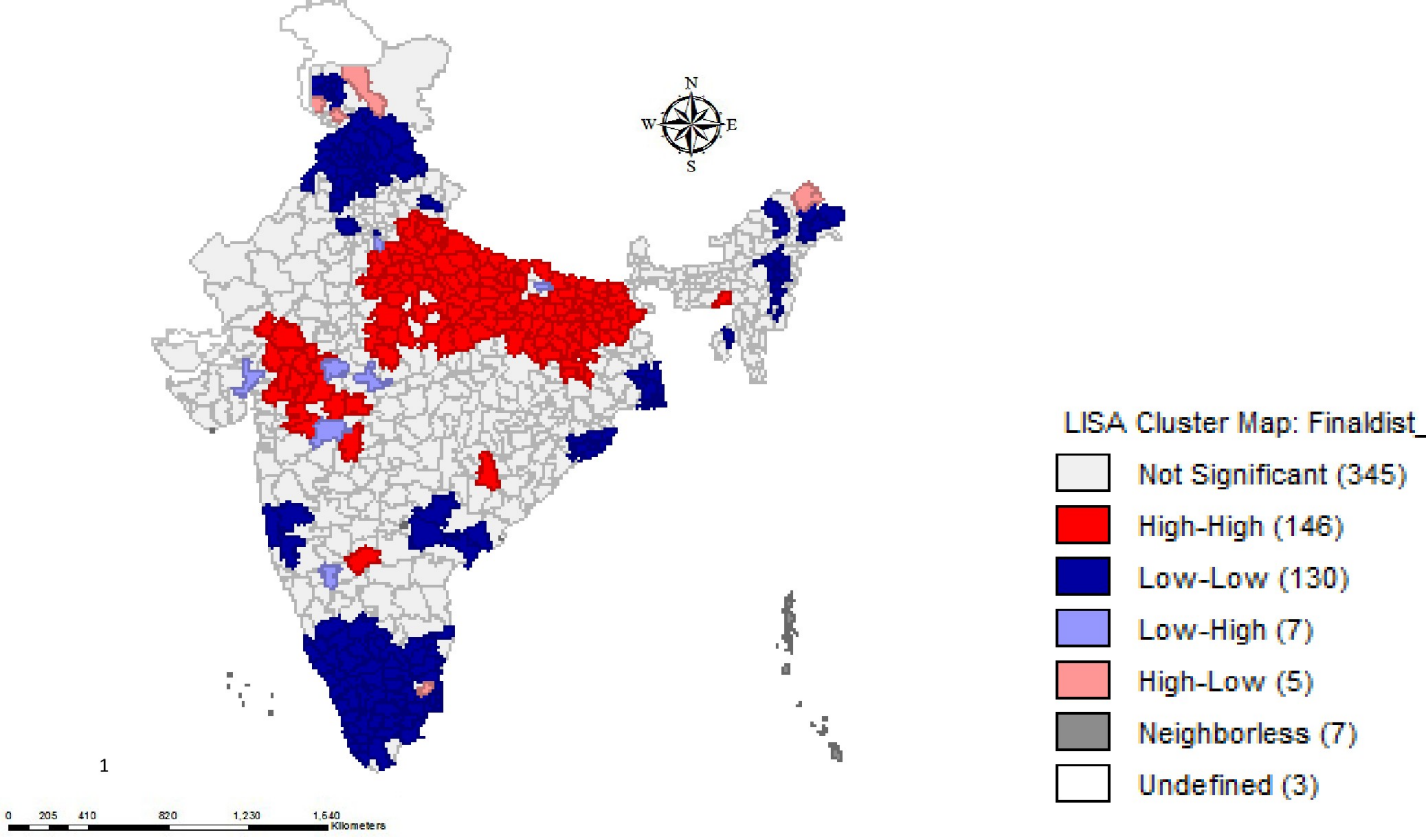
Univariate LISA map for spatial clustering of stunting across districts of India, 2015–2016.

The LISA cluster map presented in Fig 1 shows that there were 146 hot spots, that is, districts with high prevalence of CS surrounded by other high-prevalence districts, mostly in the states of Bihar, Jharkhand, Madhya Pradesh, and Uttar Pradesh. It also shows that there were 130 cold spots, that is, districts with low prevalence of CS surrounded by other low-prevalence districts, largely in the states of Arunachal Pradesh, Himachal Pradesh, Kerala, Punjab, Tamil Nadu, and West Bengal.

### Spatial heterogeneity in potential risk and protective factors of childhood stunting

**Fig 2:**
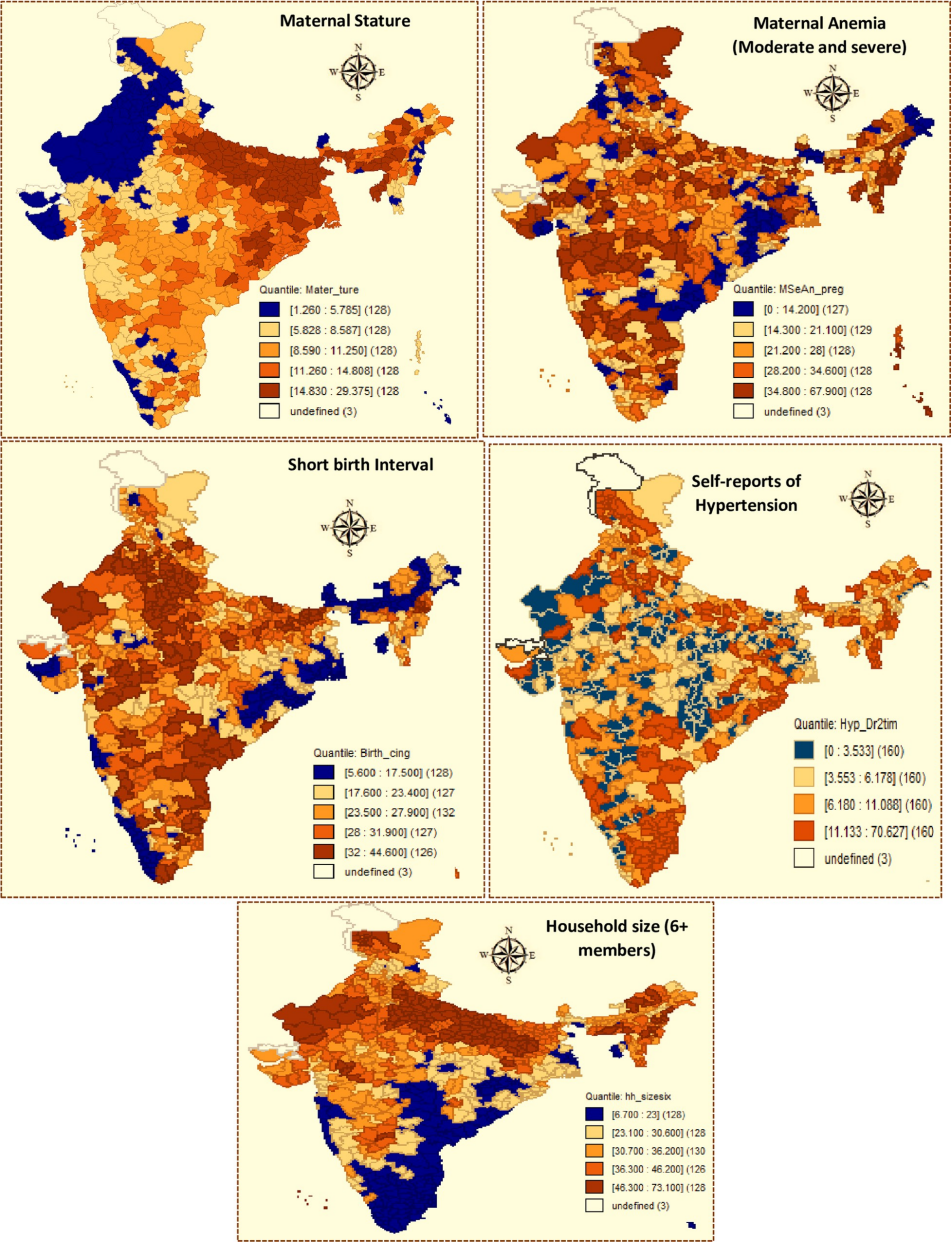
Quintile maps for explanatory variables.

As with the prevalence of CS, there were noticeable variations in the mean values of the explanatory variables across districts (Table 1 and Fig 2). Nationally, 10 percent of women who gave birth in the last five years were of short stature, that is, less than 145-centimeter height. The proportion of short-statured mothers was 15–29 percent in the districts in the top quintile (128 districts) and most of these districts were located in the states of Assam, Bihar, Odisha, and Uttar Pradesh. The prevalence of moderate or severe anaemia among pregnant women aged 15–49 stood at 25 percent nationally, and it was as high as 35–68 percent in 128 districts in the top quintile spread across the country. Short birth intervals, that is, <24 months, continued to characterize a substantial proportion of births; a quarter of non-first births in the five years preceding the 2015–16 NFHS occurred within 24 months of the preceding birth nationally, and the prevalence of closely spaced births was as high as 32–45 percent in about 126 districts, mostly in the states of Andhra Pradesh, Maharashtra, Madhya Pradesh, Rajasthan, and Uttar Pradesh. Nationally, nine percent of women aged 15–49 were told by a doctor or health professional on two or more occasions that they have hypertension or high blood pressure, with a high concentration of self-reported prevalence of hypertensive disorders among women in Punjab, Tamil Nadu, Puducherry, and the northeastern states.

Nationally, 34 percent of the households contained more than six members and this proportion ranged between 46 percent and 73 percent in the 128 top quintile districts, mostly in Bihar, Rajasthan, and Uttar Pradesh. Educational attainment among females remains low in India—just 35 percent of women aged 15–49 had completed 10 or more years of schooling, and this proportion was as low as 9–22 percent in 125 districts in the bottom quintile, mostly in the central, northern, and eastern states. The quintile distribution of households belonging to the poorest wealth quintile shows that such households were concentrated in the states of Chhattisgarh, Maharashtra, and Uttar Pradesh. Improved drinking water was accessible to 88 percent of households, with 256 districts reporting near universal access to improved drinking water, that is, 95 percent or more households having such access, mostly in Bihar, Punjab, Tamil Nadu, Uttar Pradesh, and West Bengal. However, despite efforts to make India open-defecation free, as many as 39 percent of households were practising open defecation, and this was prevalent in as many as 67–90 percent of households in 128 districts, largely in the states of Bihar, Madhya Pradesh, Odisha, and Uttar Pradesh.

The breastfeeding and complementary feeding practices of infants and young children were far from satisfactory. Only 45 percent of mothers aged 15–49 had breastfed their newborn/s within an hour of delivery, and districts with low levels of early initiation of breastfeeding were concentrated in the states of Madhya Pradesh, Odisha, and the northeastern states. About a quarter of children aged 6–23 months received adequately diverse diet on the last day, that is, consumed items from more than four food groups during the previous day of the survey, and the proportion of children who had minimum dietary diversity ranged between 31 percent and 80 percent in districts that scored in the top quintile, mostly in Arunachal Pradesh, Kerala, Meghalaya, Tamil Nadu, and West Bengal.

Some eight percent of children under five years of age had experienced diarrhoea in the two weeks preceding the 2015–16 NFHS. The prevalence of diarrhoea ranged from 12 percent to 45 percent in the 128 top quintile districts, concentrated in the states of Madhya Pradesh, Odisha, and Uttar Pradesh.

The extent to which children and women benefitted from nutrition-specific interventions presented a mixed picture. Access to health and nutrition information, as measured by a proxy indicator of percentage of women aware of oral rehydration salt (ORS) was high, with 84 percent reporting awareness of ORS. However, the reach and uptake of several other interventions were limited:for example, just 31 percent of mothers who delivered in the last five years had consumed 100 or more doses of iron and folic acid supplements; the consumption levels were as low as 1–12 percent in 126 districts in the bottom quintile, mainly in Arunachal Pradesh, Bihar, Rajasthan, and Uttar Pradesh. Three-fifths of children aged 9–59 months had received vitamin A supplements in the six months preceding the survey; the intake stood at 13–45 percent in 127 districts in the bottom quintile, mostly in Bihar, Nagaland, Rajasthan, and Uttar Pradesh. Mothers of 49 percent of children under six years had received food supplementation through the Integrated Child Development Services programme during pregnancy or lactation. Food supplementation to mothers was particularly limited (30% or less) in several districts of the central, northern, and northeastern regions. Access to social protection schemes was also limited: just 26 percent of households were covered by any health insurance schemes, and health insurance coverage was as low as six percent or less in the bottom quintile districts, mainly in Assam and Uttar Pradesh.

Nationally, population density in 2011 was 936 persons per square kilometer; the population density was as low as 183 or fewer persons/square kilometer in 127 districts. Development indicators—urbanization and electrification—also showed a mixed picture. About a quarter of the population was residing in urban areas in 2011 nationally; the level of urbanization was over 40 percent in 124 districts, mostly in Gujarat, Kerala, Maharashtra, and Tamil Nadu. Electrification, as measured by percentage of villages electrified, stood at 66 percent nationally in 2011; however, there were 128 districts in which no more than one-third of villages were electrified, mostly in Assam, Bihar, Jharkhand, and Uttar Pradesh. Finally, data on climatic conditions from 2011 show that the average extreme temperature recorded was 40° Celsius nationally.

### Bivariate association between childhood stunting and potential predictors

**Fig 3:**
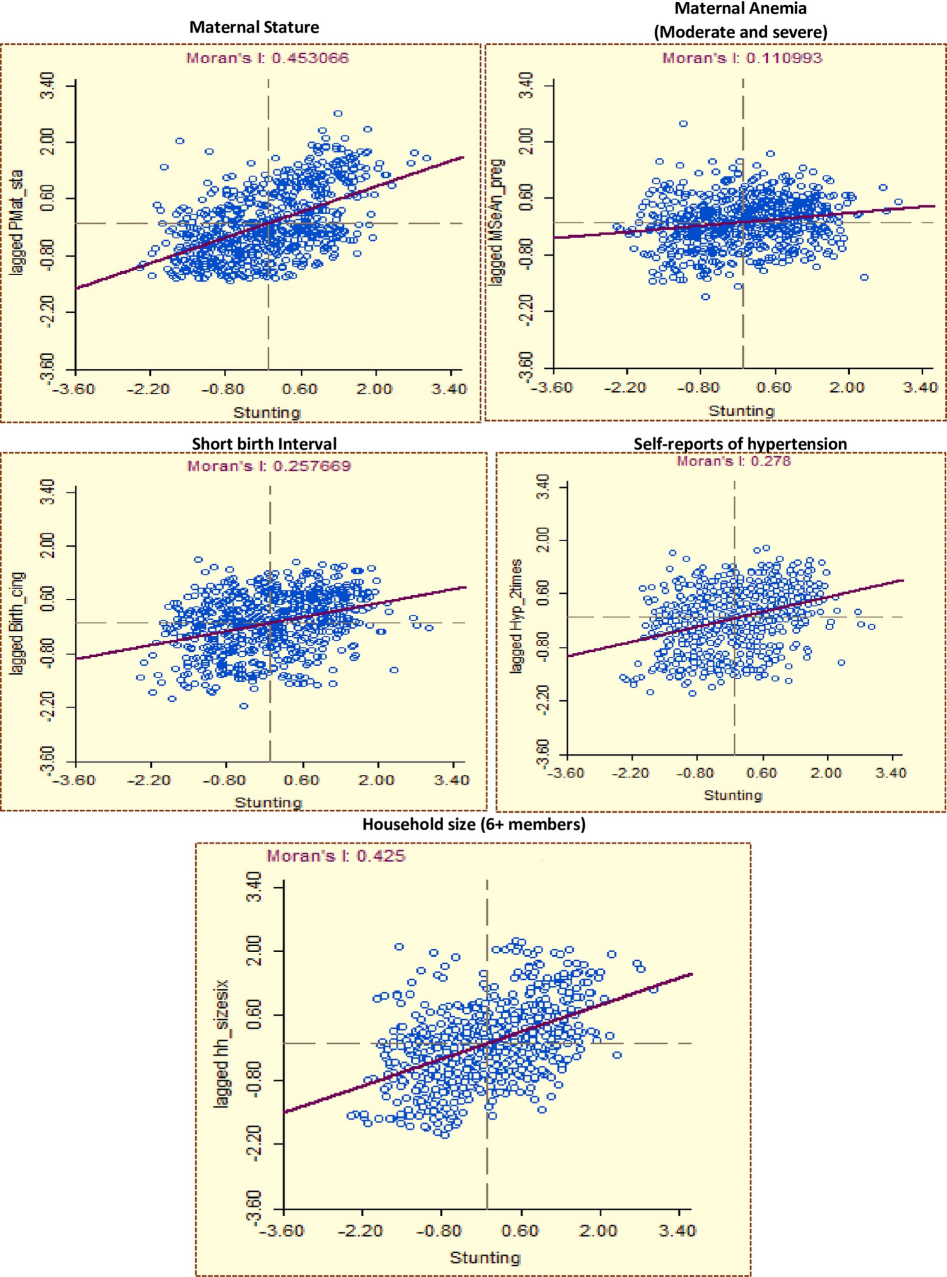
Bivariate spatial association between childhood stunting and explanatory variables.

Fig 3 presents Moran’s I scatter plots exploring the bivariate association between CS and potential predictors.

Of the maternal- and home-environment-related variables, a positive spatial association was observed between CS and maternal short stature (Moran’s I = 0.453), short birth interval (Moran’s I = 0.258), self-reported prevalence of elevated blood pressure among women (Moran’s I = 0.278), household size (Moran’s I = 0.425), and open defecation (Moran’s I = 0.526). In comparison, a negative spatial correlation was observed between CS and female education (Moran’s I = -0.467). The bivariate Moran’s I value was small for several variables such as maternal anaemia (Moran’s I = 0.111), household poverty (Moran’s I = 0.134), and access to improved drinking water sources (Moran’s I = 0.072).

Of indicators related to infant and child feeding practices and infections, findings show an inverse spatial association between CS and early initiation of breastfeeding (Moran’s I = -0.325), minimum dietary diversity (Moran’s I = -0.444), and the prevalence of diarrhoea in children (Moran’s I = 0.244).

An inverse spatial association was observed between CS and nutrition-specific intervention-related indicators such as awareness of ORS (Moran’s I = -0.250), vitamin A supplementation for children (Moran’s I = -0.248), pregnant women’s consumption of IFA tablets (Moran’s I = -0.459), and household access to health insurance schemes (Moran’s I = -.0.301). Although an inverse association was observed for food supplementation given to women during pregnancy and to lactating mothers as well, the Moran’s I value was small (-0.153).

An inverse spatial correlation was observed with village electrification (Moran’s I = -0.521) and urbanization (Moran’s I = -0.218). Finally, extreme temperature was positively associated with CS (Moran’s I = 0.372).

### Determinants of childhood stunting

Results from the spatial error model (SEM) are presented in Table 3. Model 1 results indicate that among maternal and home environment factors, maternal short stature (coefficient 0.590; p<0.000), short birth interval (coefficient 0.109; p<0.002), self-reported prevalence of elevated blood pressure among women (coefficient 0.014; p<0.032), household size (coefficient 0.213; p<0.000), household poverty (coefficient 0.014; p = 0.051), and open-defecation practice (coefficient 0.099; p<0.000) were positively associated with the prevalence of CS. In contrast, female education (coefficient -0.120; p<0.000) and access to improved drinking water sources (coefficient -0.048; p<0.023) were inversely correlated with CS. However, we found no association between CS and maternal anaemia in Model 1.

**Table 3:** Unadjusted and adjusted coefficients for the spatial regression analysis of the risk factors of childhood stunting.

Variables	Model 1: Household and Family factors Coeff. (S.E, p-value)	Model 2: Complementary and breastfeeding practices and infections Coeff (S. E, p-value)	Model 3: Contextual and System factors Coeff (S. E, p-value)	Model 4: Full model Coeff (S. E, p-value)
Maternal stature	**0.59**			**0.601**
	(0.068, 0.000)			(0.069, 0.000)
Maternal anaemia	0.004			0.012
	(0.018, 0.800)			(0.017, 0.511)
Short birth interval	**0.109**			**0.119**
	(0.035, 0.002)			(0.036, 0.001)
Self-reported prevalence of elevated blood pressure	**0.014**			**0.0164**
	(0.014, 0.032)			(0.015, 0.028)
Household size	**0.213**			**0.192**
	(0.028, 0.000)			(0.031, 0.000)
Female education	**-0.12**			**-0.130**
	(0.028, 0.000)			(0.031, 0.000)
Household poverty	**0.014**			**0.031**
	(0.021, 0.051)			**(0.023, 0.015)**
Access to improved drinking water sources	**-0.048**			**-0.049**
	(0.021, 0.023)			**(0.021,0.025)**
Open defecation	**0.099**			**0.088**
	(0.016,0.000**)**			**(0.017,0.000)**
Early initiation of breastfeeding		-0.026		-0.001
		(0.022, 0.242)		(0.018, 0.94)
Dietary diversity		**-0.151**		**-0.158**
		(0.026, 0.000)		(0.049, 0.001)
Prevalence of diarrhoea in children		0.053		-0.033
		(0.056, 0.346)		(0.046, 0.474)
Urbanization			**-0.011**	
			(0.017,0.044)	
Awareness of nutrition-promoting actions			**-0.009**	-0.001
			(0.029, 0.003)	(0.026, 0.96)
Consumption of 100 or more IFA tablet by pregnant women			**-0.061**	**-0.089**
			(0.022, 0.006)	(0.022, 0.030)
Micronutrient intake among children			**-0.043**	-0.016
			(0.021, 0.003)	(0.018, 0.37)
Food supplementation through ICDS			**-0.061**	-0.003
			(0.019, 0.001)	(0.017, 0.856)
Access to health insurance schemes			-0.009	-0.010
			(0.019, 0.624)	(0.015, 0.542)
Population density			0.000	0.001
			(0.001, 0.480)	(0.001, 0.785)
Access to electricity			**-0.127**	-0.005
			(0.018, 0.000)	(0.030, 0.763)
Extreme temperature			**0.137**	**0.082**
			(0.056, 0.013)	**(0.049, 0.044)**

Values in parentheses show standard error and p values.

Model 2 results show that among variables related to breastfeeding and complementary feeding practices and infections, dietary diversity among children was inversely associated with the prevalence of CS (coefficient -0.151, p<0.000). Other indicators—early initiation of breastfeeding and prevalence of diarrhoea—though significant in the bivariate analysis lost significance in the multivariate analysis.

Model 3 that explored the association of contextual factors shows that nutrition-specific interventions, such as awareness of nutrition-promoting actions (coefficient -0.009; p = 0.003), iron and folic acid supplementation during pregnancy (coefficient -0.061, p = 0.006), vitamin A supplements for children (coefficient -0.043, p = 0.003), and food supplementation for pregnant women and lactating mothers through the Integrated Child Development Services (coefficient -0.061, p = 0.001), were inversely correlated with CS. However, access to health insurance schemes was not correlated with CS. Among demographic and development factors, village electrification and urbanization were inversely related to CS (coefficient -0.127, p = 0.000 for village electrification; coefficient -0.011, p = 0.044 for urbanization). No association was observed between population density and CS. Extreme temperature was positively correlated (coefficient -0.137, p = 0.013).

Findings presented in Model 4 show that most maternal- and home-environment-related factors—maternal short stature, short birth interval, self-reported prevalence of elevated blood pressure among women, household size, female education, household poverty, household access to improved drinking water sources, and open-defecation practice—remained significant in the full model. While maternal short stature, short birth interval, self-reported prevalence of elevated blood pressure among women, large household size, household poverty, and open-defecation practices were associated with increased risk of CS, female education and household access to improved drinking water sources were associated with reduced risk of CS. Similarly, of indicators related to breastfeeding and complementary feeding practices, minimum dietary diversity remained significantly associated with reduced risk of CS in the full model. Of the contextual factors, iron and folic acid supplementation during pregnancy was associated with reduced risk of CS. Extreme temperature was correlated with increased risk of CS. The effect of a number of contextual factors—awareness of nutrition-promoting actions, micronutrient intake among children, food supplementation through ICDS for pregnant women and lactating mothers, and village electrification—was attenuated in the full model.

## Discussion

The study findings reiterate that CS continues to be high in India, with several hot spot states and districts, and that children from the central and eastern region of the nation, namely, Bihar, Jharkhand, Madhya Pradesh, and Uttar Pradesh are particularly vulnerable. These findings concur with observations of previous studies on spatial heterogeneity of CS in India [13,17,18,22]. Our analysis has identified six risk factors—maternal short stature, large household size, closely spaced births, prevalence of hypertension among women, household poverty, open defecation, and extreme temperature—and four protective factors—female education, access to improved drinking water, dietary diversity among children, and iron and folic acid supplementation during pregnancy.

Maternal short stature emerged as the strongest risk factor for CS even after adjusting for a range of proximal and contextual factors, with the risk of CS higher by 0.57 with one unit increase in the proportion of short-statured mothers. This finding is consistent with observations from studies from India and multi-country studies [12,18,34–37] and reflects the intergenerational transmission of disadvantage.

Closely spaced births and large household size were the two other powerful risk factors for CS in the full model, with the risk of CS higher by 0.11 and 0.19 with one unit increase in the prevalence of closely spaced births and large household size, respectively. Short birth intervals are associated with a higher risk of maternal anaemia, preterm births, and low birthweight, and the causal pathways may include maternal depletion, competition for maternal attention and household resources, and cross-infection [11,38–40].

Household-level poverty and open defecation were two other risk factors identified in our analysis, with the risk of CS higher by 0.03 with one unit increase in the proportion of households belonging to the lowest wealth quintile and by 0.08 with one unit increase in the proportion of households practicing open defecation. These findings concur with findings of several previous studies in India [14,17,18,21–23].

To the best of our knowledge, none of the previous studies on CS in India has examined the association between CS and metabolic syndromes in adulthood. The NFHS-4 not only measured the blood pressure of women aged 15–49 and men aged 15–54, but also probed them on whether they were told by a physician or other health professional on two or more occasions that they have high blood pressure, thereby, giving a unique opportunity to examine the relationship between CS and metabolic syndromes in adulthood. We used the proxy measure of the proportion of women who gave birth in the five years preceding the survey and who reported that they were told by a health professional that they had high blood pressure. Our analysis shows that the risk of CS was higher by 0.02 with one unit increase in the self-reported prevalence of hypertension among mothers. Newborns whose mother had hypertension during pregnancy tend to have insufficient supply of nutrition during pregnancy and reduced level of insulin-like growth factor 1 (IGF-1), an important stimulus for fetal linear growth and weight gain [41–43].

Study findings also highlight the risk of CS associated with climate change, especially with extreme temperature—a finding observed in a recent macro-level study in India [23]. Increased frequency of droughts and flooding associated with climate change may reduce food availability and dietary diversity and may increase the prevalence of infectious diseases such as diarrhoea or malaria [44].

The strongest protective factor against CS identified in the study was female education, with the risk of CS lower by 0.12 by one unit increase in the proportion of women of reproductive ages who had completed secondary education. The reduced risk of CS associated with female (maternal) education has been consistently documented in Indian and global literature [15,16,18,44, 45]. Several pathways indicative of the positive effects of maternal education in reducing CS have been put forward, including increased earnings, access to health and nutrition information, receptivity to modern medicine, and enhanced agency and social networks [44].

In India, the current evidence on the contributions of nutrition-specific interventions in reducing CS is mixed. While a couple of studies, for example, observed an inverse association between iron and folic acid supplementation during pregnancy and CS [46,47], a few others observed no such relationship [12,18]. No relationship, likewise, was observed between CS and vitamin A supplementation for children [18] and food supplementation through the Integrated Child Development Services [12,19]. Our analysis, however, highlights the possible reduction in CS that can be achieved by increasing the reach of such interventions, particularly in vulnerable geographies. The association between iron and folic acid supplementation during pregnancy was consistent and robust in the partial model as well as in the full model, with increased uptake of iron and folic acid supplementation associated with reduced prevalence of CS. A systematic review reported that iron or iron and folic acid supplementation during pregnancy can reduce anaemia at term, iron-deficiency anaemia, and the incidence of low birthweight [48]. The association between other nutrition-specific interventions—vitamin A supplements for children under five years and food supplementation for women during pregnancy or mothers during lactation—and CS was also significant in the partial model, although the effect of both these indicators was attenuated and not significant in the full model, perhaps because of less than adequate direct investment in these programmes [49,50] or because of the large number of explanatory variables used in this analysis.

Two other protective factors identified in the study and also observed in previous studies include dietary diversity among children and household access to improved drinking water [51–54]. Improved feeding practices, particularly consumption of adequately diversified food, can lead to improved intake of nutrients, which will improve nutrition among children [51,55].

### Potential limitations

The limitations of the study are acknowledged. One of the limitations of this study is the temporal mismatch among the covariates used in the analysis. While data points for a few covariates such as access to electricity and climate factors referred to 2011, others referred to 2015–2016. Given the cross-sectional nature of the survey data, bivariate and multivariate association pattern observed in the study does not indicate causation between the exposure and response variables. Apart from that, the study also acknowledges the fact that there is non-correspondence in timing of exposure of CS and some of the explanatory variables such as dietary diversity and early initiation of breastfeeding. Additionally, results for areas on the peripherals (borders) of the current analysis are likely biased, though it is difficult to say in which direction, as they have missing “neighbours” which may or may not be similar to the region in question.

## Conclusions and policy implications

The study presents a comprehensive picture of proximal and contextual determinants of CS in India and expands the current evidence base. The study has identified several risk and protective factors, some of which are extensively explored in earlier studies (proximal factors) and some of which are not so well explored (contextual factors) in the Indian context. The study findings have several policy implications.

The study findings call for effective implementation of various components of maternal, newborn, and childcare, for example, pre-conception care, essential tests and check-ups that are a part of antenatal care, and complication readiness stipulated in the Reproductive, Maternal, Neonatal and Child Health + Adolescents (RMNCH+A) programme in India. Although the RMNCH+A programme seeks to provide continuum of care that focuses on the pre-pregnancy stage and extends to the childcare phase of the life cycle [56], pre-conception care remains largely neglected [57]. In particular, programmes have neglected adolescent girls and young women (and young couples) in the period prior to first pregnancy [58]. The NFHS-4 reports that just 15 percent of 15–19-year-old women compared with 36 percent of 20–29-year-old women and 11 percent of never married women compared with 28 percent of currently married had interacted with a community-level health worker in the three months preceding the interview [2], highlighting further the current lack of focus on pre-conception care. The study findings that maternal short stature was the strongest risk factor for CS call for increasing the investment in pre-conception care and sensitizing health workers about the relationship between maternal stature and CS and the importance of paying special attention to short-statured women. Similarly, findings on the relationship between prevalence of hypertension among women and CS argue for efforts to universalize essential check-ups and tests that are part of antenatal care—checking blood pressure and testing urine, for example. Although checking blood pressure is included in the package of comprehensive antenatal care, and the healthcare providers are expected to inform pregnant women about complications that can arise from hypertensive disorders in pregnancy, NFHS-4 data show that only 75 percent of women who had given birth in the five years preceding the survey had their blood pressure tested at least once during the last pregnancy and just 37 percentwere informed about complications related to hypertensive disorders during their antenatal visits [2], which highlight missed opportunities.

The study findings call for improved attention to providing contraceptive counselling and services to young people. Several studies, including the most recent NFHS, have highlighted huge unmet need for contraceptives for spacing among married young women [2,59,60]. There is also evidence that substantial proportions of married adolescent girls and young women in India prefer to delay their first pregnancy, although very few are able to act on their desires for a variety of reasons [59–61]. Therefore, contraceptive information should be provided to unmarried adolescents and newly married young people, using multiple platforms, including the school health programme, the peer educators of the Rashtriya Kishor Swasthya Karyakram, and digital media platforms. Contraceptive methods that are appropriate for young people should be made easily available and accessible to them. Healthcare providers, including frontline workers, need to be sensitized about married young people’s desire to delay first pregnancy, their contraceptive needs, and the importance of reaching out to them before they experience the first pregnancy.

A review article by the Lancet Nutrition Interventions Review Group and the Maternal and Child Nutrition Study Group notes that a fifth of the existing burden of stunting can be averted if selected nutrition-specific interventions can be scaled up to 90 percent coverage [62]. While a number of nutrition-specific interventions are implemented in India, their reach remains limited and uneven—for example, the consumption of iron and folic acid among women with a live birth in the five years preceding the interview ranged from 14 percent among women belonging to the poorest wealth quintile to 48 percent among women belonging to the richest wealth quintile [2]. The reach of adolescent nutrition interventions also remains low and uneven in India [59,60]. Addressing these lacunae is critical in helping the nation achieve the goals set by the NNM. This would require paying attention to both demand- and supply-side barriers, including ensuring universal registration of pregnant women, addressing concerns about side effects and perceived lack of need, as seen in the consumption of iron and folic supplements, and improving the distribution, including community-based delivery of nutrition-specific intervention, including community-based delivery [47,63–66]. Findings highlight the need for implementing programmes to promote the intake of healthy foods from an early age. Context-specific nutrition information must be disseminated in a simple, understandable, and accessible manner to children, adolescents, and their parents, and these efforts must target vulnerable population groups and geographies [67].

Findings that female education was the strongest protective factor in our analysis emphasize the need for universalizing quality primary and secondary education that is inclusive and equitable. Programmes to improve the schooling situation in the country, for example, through the Sarva Shiksha Abhiyan and the Rashtriya Madhyamik Shiksha Abhiyan, need to be effectively implemented, along with special efforts to reach the most disadvantaged groups. Initiatives are also needed to create a school climate that is learning friendly. The Government of India’s initiative for vocationalization of secondary and higher secondary education must be expanded, and the content of such courses should be tailored to help children gain market-driven job skills. Campaigns like Beti Bachao Beti Padhao must raise parental aspirations for their children’s education and advocate greater parental involvement and investment in their children’s education. Finally, ‘second-chance’ programmes for adolescents who had never been to school or discontinued schooling before completing primary or secondary education are also warranted.

## Supporting information

S1 Table Difference in outcome variable and selected characteristics between districts that were included in the analysis and those that were excluded. (DOCX)

S2 Table Diagnostics analysis of degree of multicollinearity in explanatory variables. (DOCX)
