# Correction: Proximal and contextual correlates of childhood stunting in India: A geo-spatial analysis

**DOI:** 10.1371/journal.pone.0241736

**Published:** 2020-10-28

**Authors:** The PLOS ONE Staff

The Funding statement is incorrect. The correct Funding statement is as follows: Research reported in this publication was initially funded by the John D and Catherine T MacArthur Foundation through a grant to the Population Council to undertake policy research and advocacy for strategic investment in maternal newborn and child health in India (G-109245-0). The content is solely the responsibility of the authors and does not necessarily represent the official views of the funding institution. The funders had no role in study design, data collection and analysis, decision to publish, or preparation of the manuscript.

Fig 2 and Fig 3 do not appear in full. The publisher apologizes for the error. Please see the full Fig 2 as S1 Fig below and the full Fig 3 as S2 Fig below.

## Supporting information

S1 Fig Quintile maps for explanatory variables. (PDF)

S2 Fig Bivariate spatial association between childhood stunting and explanatory variables. (PDF)
