# Supplementary material for: Proximal and contextual correlates of childhood stunting in India: A geo-spatial analysis
Source: PLoS One. 2020 Aug 20;15(8):e0237661. doi: 10.1371/journal.pone.0237661 (PMC7446880; doi:10.1371/journal.pone.0237661)
Supplement: S1 Table — (DOCX) [file pone.0237661.s001.docx]

**S1 Table. Difference in outcome variable and selected characteristics between districts that were included in the analysis and those that were excluded**

| **Outcome variable and selected characteristics** | **District excluded from the analysis** | **Districts included in the analysis** | **Mean difference** |
| --- | --- | --- | --- |
| % of women aged 15–49 who had completed 10 or more years of schooling (%) | 32.3 | 34.6 | -2.3 |
| Bottom wealth quintile (%) | 31.7 | 23.5 | 8.2*** |
| Urban (%) | 19.7 | 26.4 | -6.7* |
| Childhood Stunting (%) | 33.9 | 35.8 | -1.9 |
| Access to electricity (%) | 64.9 | 65.5 | -0.5 |
| Social group (% SC\ST) | 73.6 | 35.0 | 38.6*** |
| Minimum Diversity Score (%) | 10.1 | 8.4 | 1.7 |
| **Total number of Districts** | **29** | **611** |  |

*** p<0.01, ** p<0.05, * p<0.1
