# Supplementary material for: Proximal and contextual correlates of childhood stunting in India: A geo-spatial analysis
Source: PLoS One. 2020 Aug 20;15(8):e0237661. doi: 10.1371/journal.pone.0237661 (PMC7446880; doi:10.1371/journal.pone.0237661)
Supplement: S2 Table — (DOCX) [file pone.0237661.s002.docx]

**S2 Table. Diagnostics analysis of degree of multicollinearity in explanatory**

**variables**

| **Explanatory variables** | **Collinearity Statistics** | |
| --- | --- | --- |
|  | **Variance inflation factor** | **Tolerance** |
| Access to electricity | 3.9 | 0.256 |
| Consumption of 100 or more IFA | 3.79 | 0.264 |
| Female education | 3.34 | 0.299 |
| Open defecation | 3.13 | 0.319 |
| Food supplementation through ICDS | 2.8 | 0.357 |
| Household size | 2.6 | 0.385 |
| Maternal stature | 2.27 | 0.441 |
| Extreme temperature | 2.01 | 0.498 |
| Early initiation of breastfeeding | 1.97 | 0.508 |
| Access to health insurance schemes | 1.91 | 0.524 |
| Dietary diversity | 1.86 | 0.538 |
| Awareness of nutrition-promoting actions | 1.81 | 0.552 |
| Micronutrient intake among children | 1.78 | 0.562 |
| Self-reported prevalence of elevated blood pressure | 1.77 | 0.565 |
| Household Poverty | 1.54 | 0.649 |
| Short birth interval | 1.53 | 0.654 |
| Prevalence of diarrhea in children | 1.3 | 0.769 |
| Population density | 1.27 | 0.787 |
| Access to improved drinking water sources | 1.17 | 0.855 |
| Maternal anemia | 1.09 | 0.917 |
| **Mean VIF** | **2.2** |  |
