# Supplementary material for: Correction: Proximal and contextual correlates of childhood stunting in India: A geo-spatial analysis
Source: PLoS One. 2020 Oct 28;15(10):e0241736. doi: 10.1371/journal.pone.0241736 (PMC7592729; doi:10.1371/journal.pone.0241736)
Supplement: S1 Fig — (PDF) [file pone.0241736.s001.pdf]

Figure 2. Quintile maps for explanatory variables

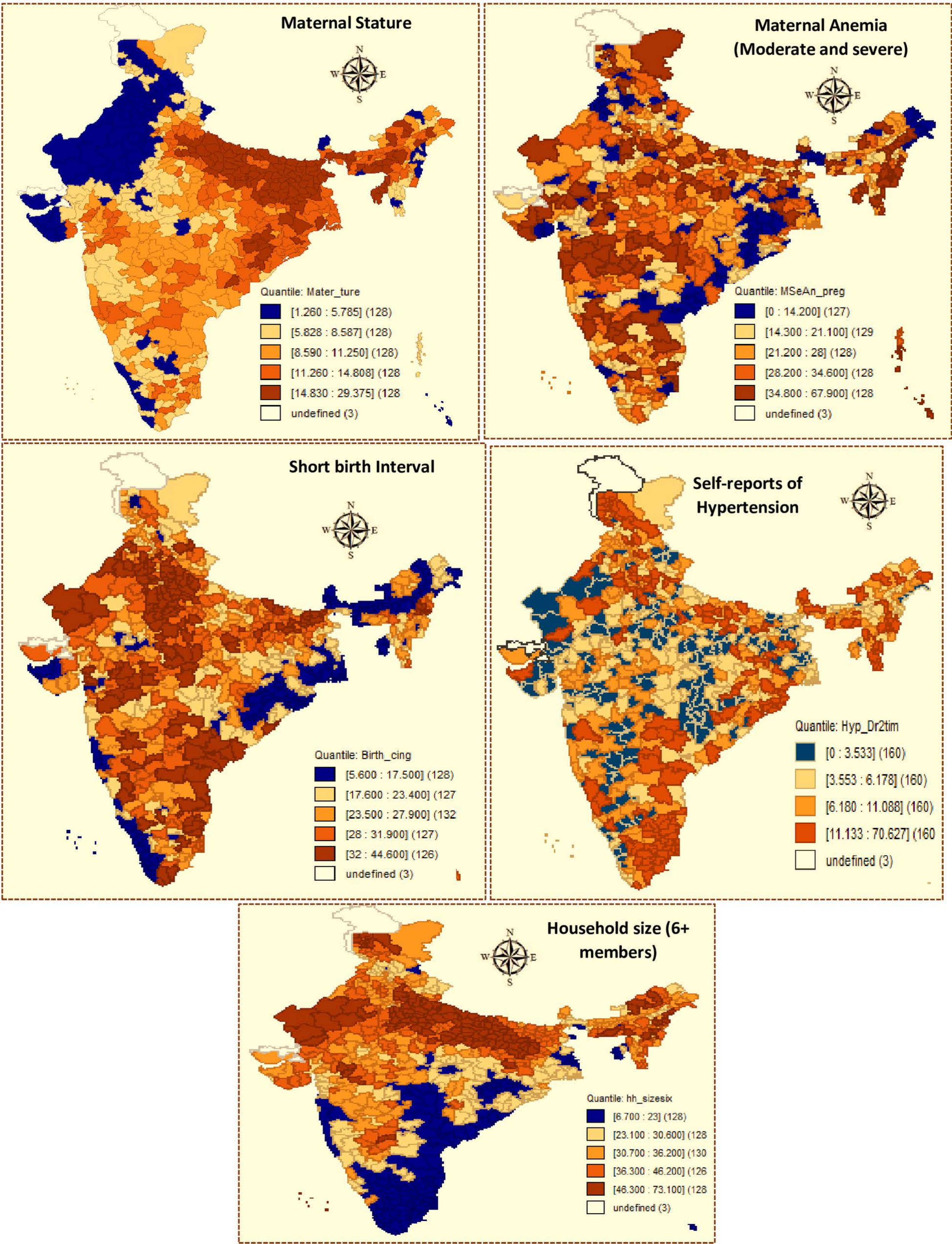

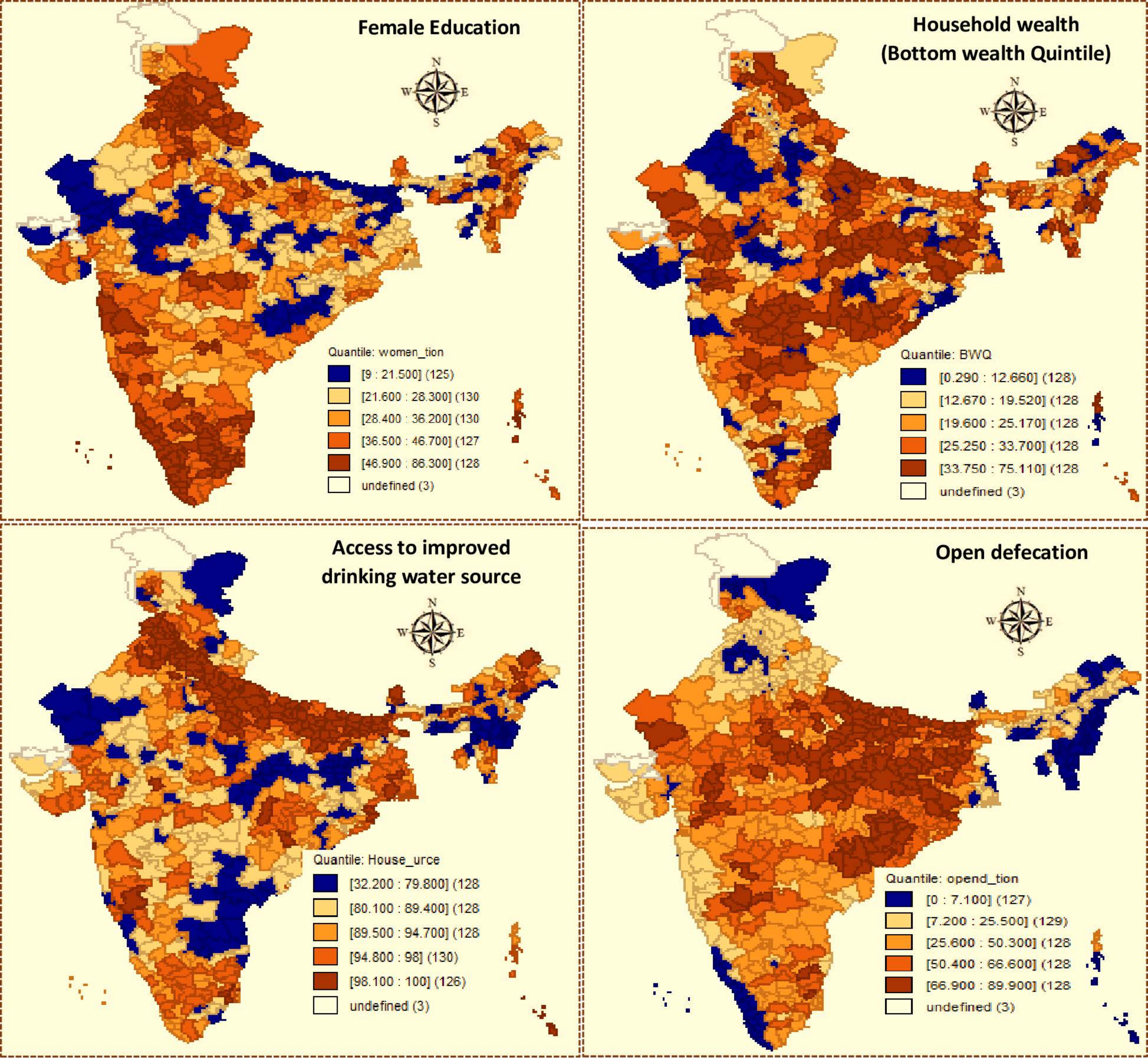

Female Education
N
W
E
S
Quantile: women_tion
[9 : 21.500] (125)
[21.600 : 28.300] (130
[28.400 : 36.200] (130
[36.500 : 46.700] (127
[46.900 : 86.300] (128
undefined (3)
Household wealth
(Bottom wealth Quintile)
N
W
E
S
Quantile: BWQ
[0.290 : 12.660] (128)
[12.670 : 19.520] (128
[19.600 : 25.170] (128
[25.250 : 33.700] (128
[33.750 : 75.110] (128
undefined (3)
Access to improved
drinking water source
N
W
E
S
Quantile: House_urce
[32.200 : 79.800] (128
[80.100 : 89.400] (128
[89.500 : 94.700] (128
[94.800 : 98] (130)
[98.100 : 100] (126)
undefined (3)
Open defecation
N
W
E
S
Quantile: opend_tion
[0 : 7.100] (127)
[7.200 : 25.500] (129)
[25.600 : 50.300] (128
[50.400 : 66.600] (128
[66.900 : 89.900] (128
undefined (3)

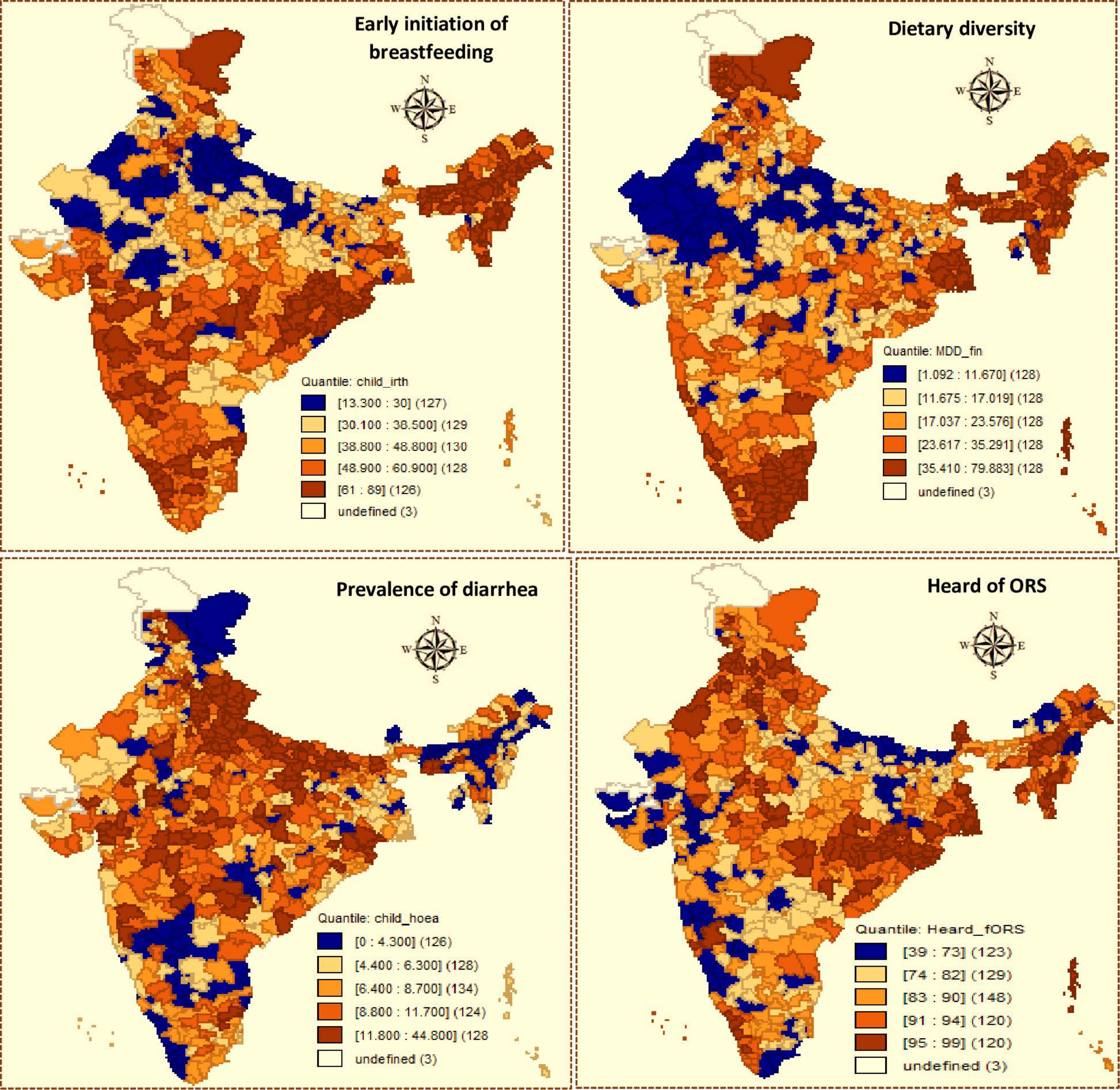
Early initiation of breastfeeding
N
W
E
S
Quantile: child_irth
[13.300 : 30] (127)
[30.100 : 38.500] (129
[38.800 : 48.800] (130
[48.900 : 60.900] (128
[61 : 89] (126)
undefined (3)
Dietary diversity
N
W
E
S
Quantile: MDD_fin
[1.092 : 11.670] (128)
[11.675 : 17.019] (128
[17.037 : 23.576] (128
[23.617 : 35.291] (128
[35.410 : 79.883] (128
undefined (3)
Prevalence of diarrhea
N
W
E
S
Quantile: child_hoea
[0 : 4.300] (126)
[4.400 : 6.300] (128)
[6.400 : 8.700] (134)
[8.800 : 11.700] (124)
[11.800 : 44.800] (128
undefined (3)
Heard of ORS
N
W
E
S
Quantile: Heard_fORS
[39 : 73] (123)
[74 : 82] (129)
[83 : 90] (148)
[91 : 94] (120)
[95 : 99] (120)
undefined (3)

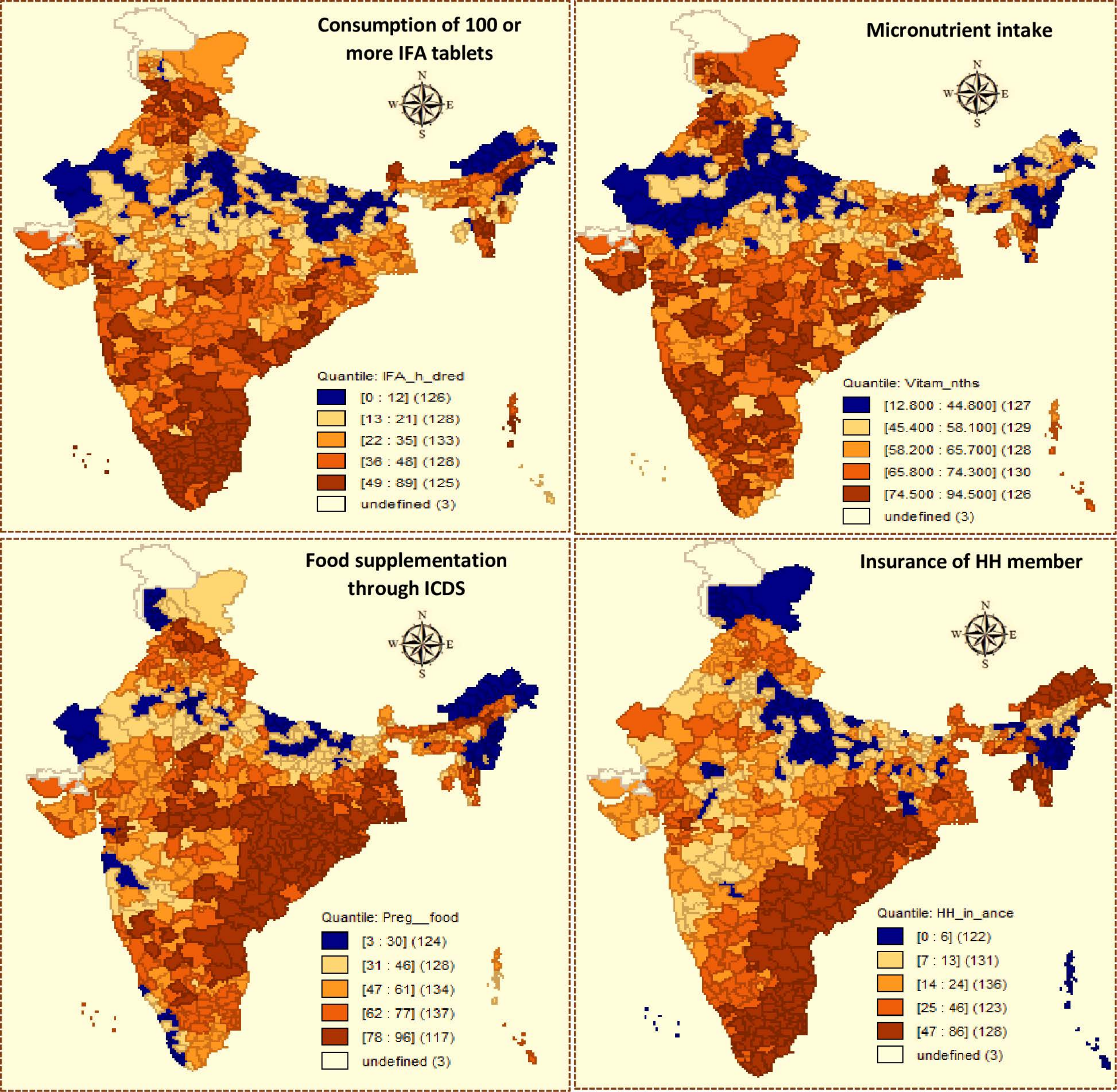
Consumption of 100 or more IFA tablets
N
W
E
S
Quantile: IFA_h_dred
[0 : 12] (126)
[13 : 21] (128)
[22 : 35] (133)
[36 : 48] (128)
[49 : 89] (125)
undefined (3)
Micronutrient intake
N
W
E
S
Quantile: Vitam_nths
[12.800 : 44.800] (127
[45.400 : 58.100] (129
[58.200 : 65.700] (128
[65.800 : 74.300] (130
[74.500 : 94.500] (126
undefined (3)
Food supplementation through ICDS
N
W
E
S
Quantile: Preg__food
[3 : 30] (124)
[31 : 46] (128)
[47 : 61] (134)
[62 : 77] (137)
[78 : 96] (117)
undefined (3)
Insurance of HH member
N
W
E
S
Quantile: HH_in_ance
[0 : 6] (122)
[7 : 13] (131)
[14 : 24] (136)
[25 : 46] (123)
[47 : 86] (128)
undefined (3)

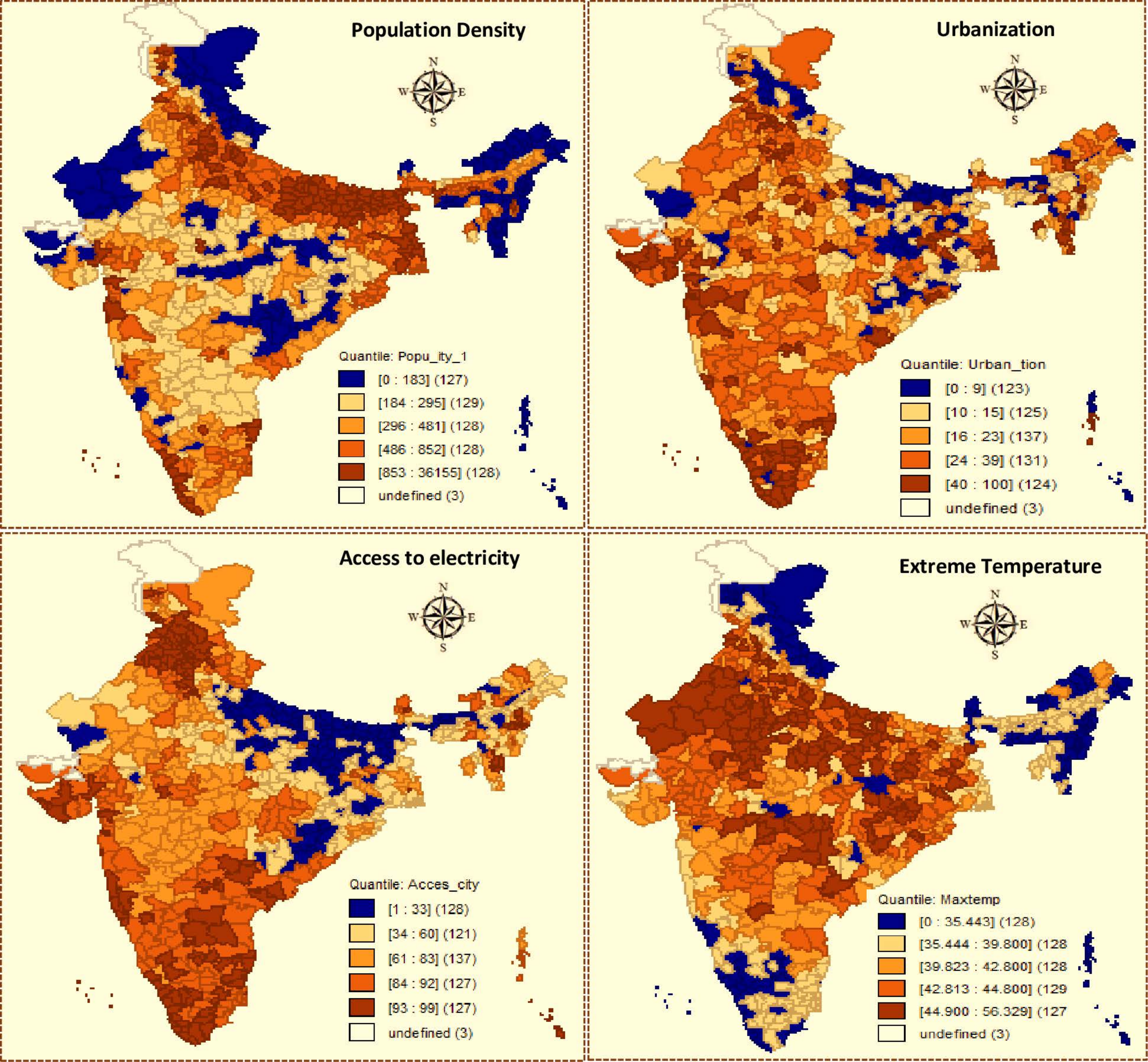
Population Density
N
W
E
S
Quantile: Popu_ity_1
[0 : 183] (127)
[184 : 295] (129)
[296 : 481] (128)
[486 : 852] (128)
[853 : 36155] (128)
undefined (3)
Urbanization
N
W
E
S
Quantile: Urban_tion
[0 : 9] (123)
[10 : 15] (125)
[16 : 23] (137)
[24 : 39] (131)
[40 : 100] (124)
undefined (3)
Access to electricity
N
W
E
S
Quantile: Acces_city
[1 : 33] (128)
[34 : 60] (121)
[61 : 83] (137)
[84 : 92] (127)
[93 : 99] (127)
undefined (3)
Extreme Temperature
N
W
E
S
Quantile: Maxtemp
[0 : 35.443] (128)
[35.444 : 39.800] (128
[39.823 : 42.800] (128
[42.813 : 44.800] (129
[44.900 : 56.329] (127
undefined (3)
