# Supplementary material for: Correction: Proximal and contextual correlates of childhood stunting in India: A geo-spatial analysis
Source: PLoS One. 2020 Oct 28;15(10):e0241736. doi: 10.1371/journal.pone.0241736 (PMC7592729; doi:10.1371/journal.pone.0241736)
Supplement: S2 Fig — (PDF) [file pone.0241736.s002.pdf]

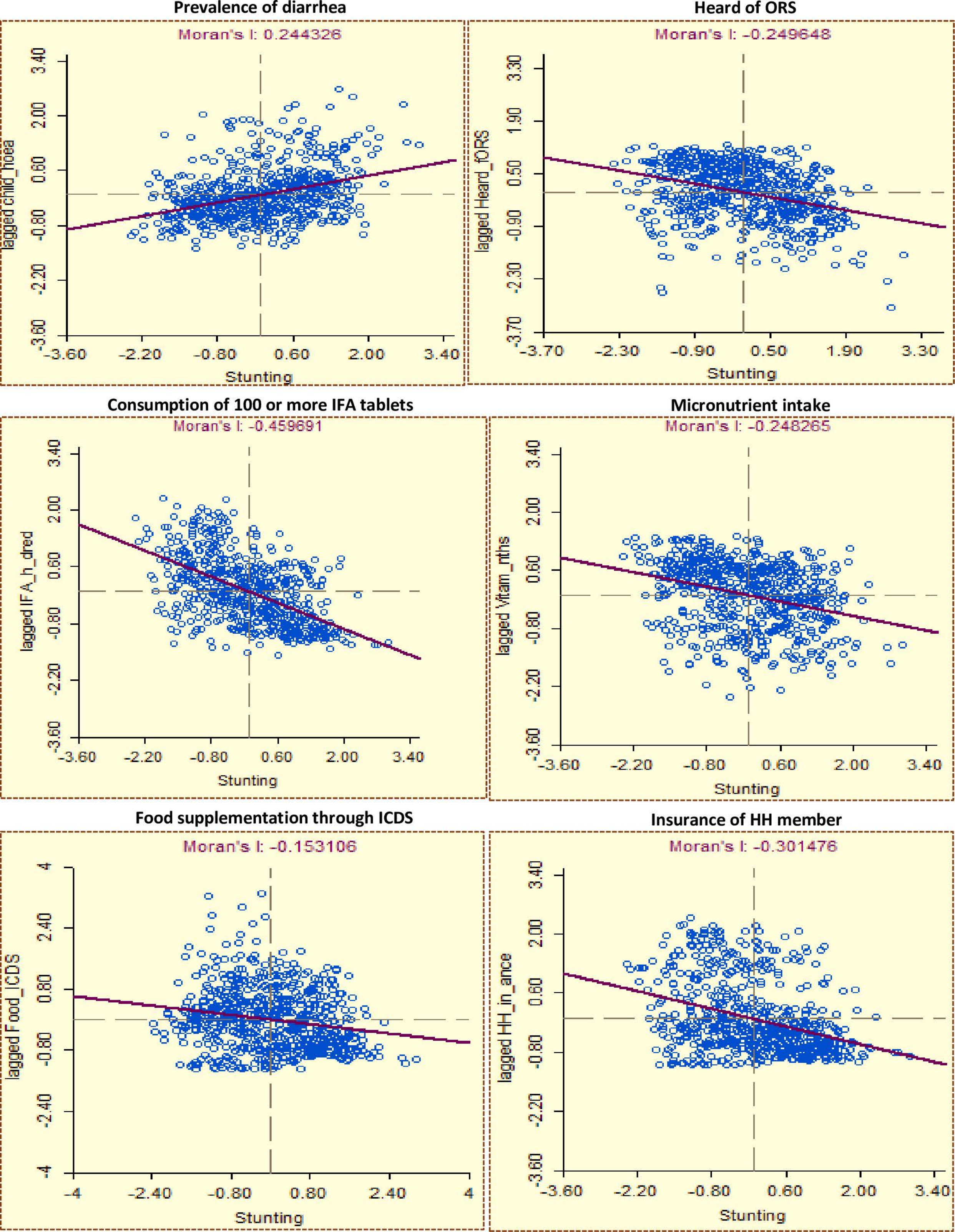

Prevalence of diarrhea
Moran's I: 0.244326
lagged child_hoea
Stunting
Heard of ORS
Moran's I: -0.249648
lagged Heard_fORS
Stunting
Consumption of 100 or more IFA tablets
Moran's I: -0.459691
lagged IFA_h_dred
Stunting
Micronutrient intake
Moran's I: -0.248265
lagged Vitam_nths
Stunting
Food supplementation through ICDS
Moran's I: -0.153106
lagged Food_ICDS
Stunting
Insurance of HH member
Moran's I: -0.301476
lagged HH_in_ance
Stunting

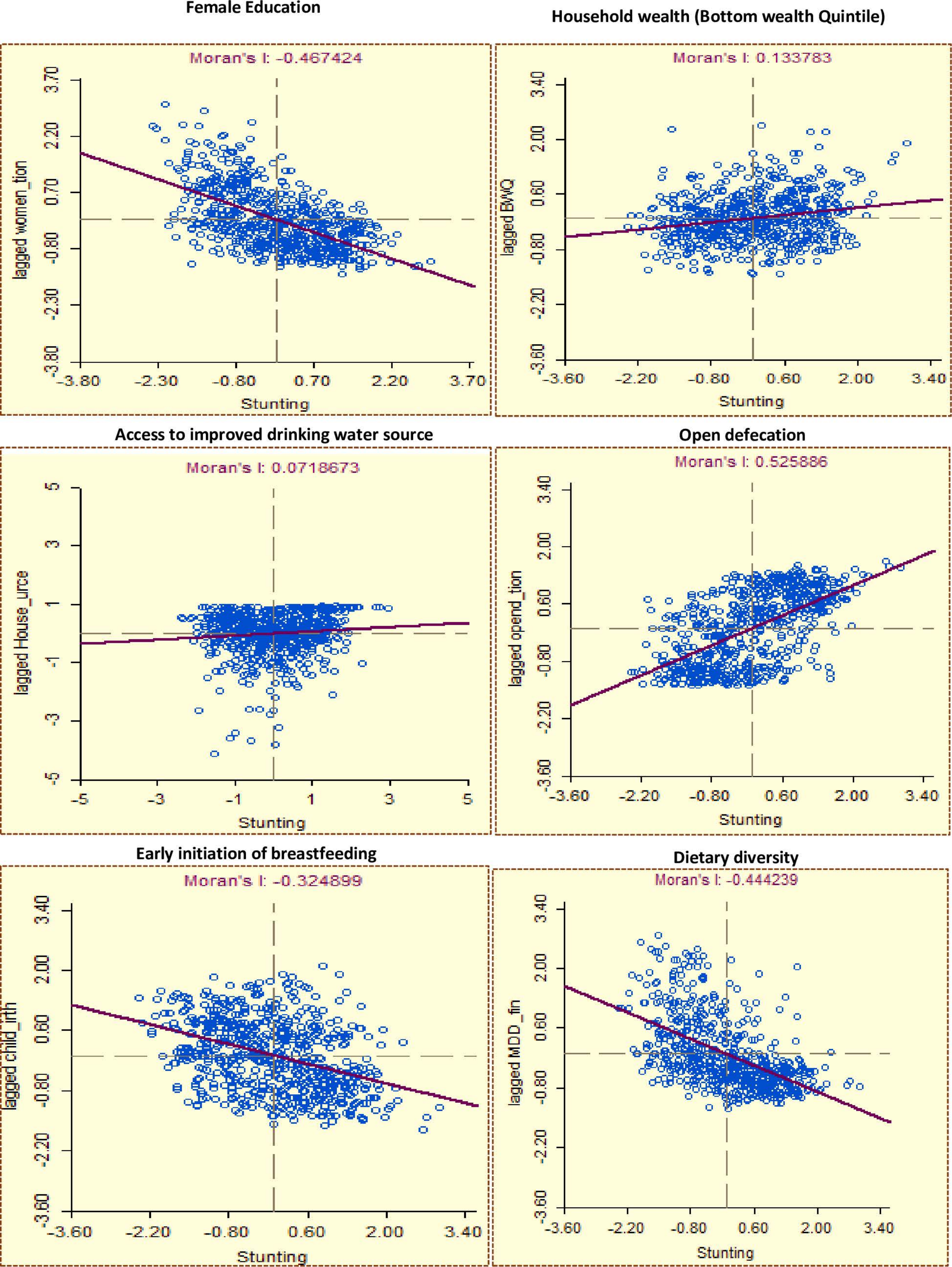
Female Education
Moran's I: -0.467424
lagged women_tion
Stunting
-3.80
-2.30
-0.80
0.70
2.20
3.70
Household wealth (Bottom wealth Quintile)
Moran's I: 0.133783
lagged BWQ
Stunting
-3.60
-2.20
-0.80
0.60
2.00
3.40
Access to improved drinking water source
Moran's I: 0.0718673
lagged House_urce
Stunting
-5
-3
-1
1
3
5
Open defecation
Moran's I: 0.525886
lagged opend_tion
Stunting
Early initiation of breastfeeding
Moran's I: -0.324899
lagged child_irth
Stunting
Dietary diversity
Moran's I: -0.444239
lagged MDD_fin
Stunting

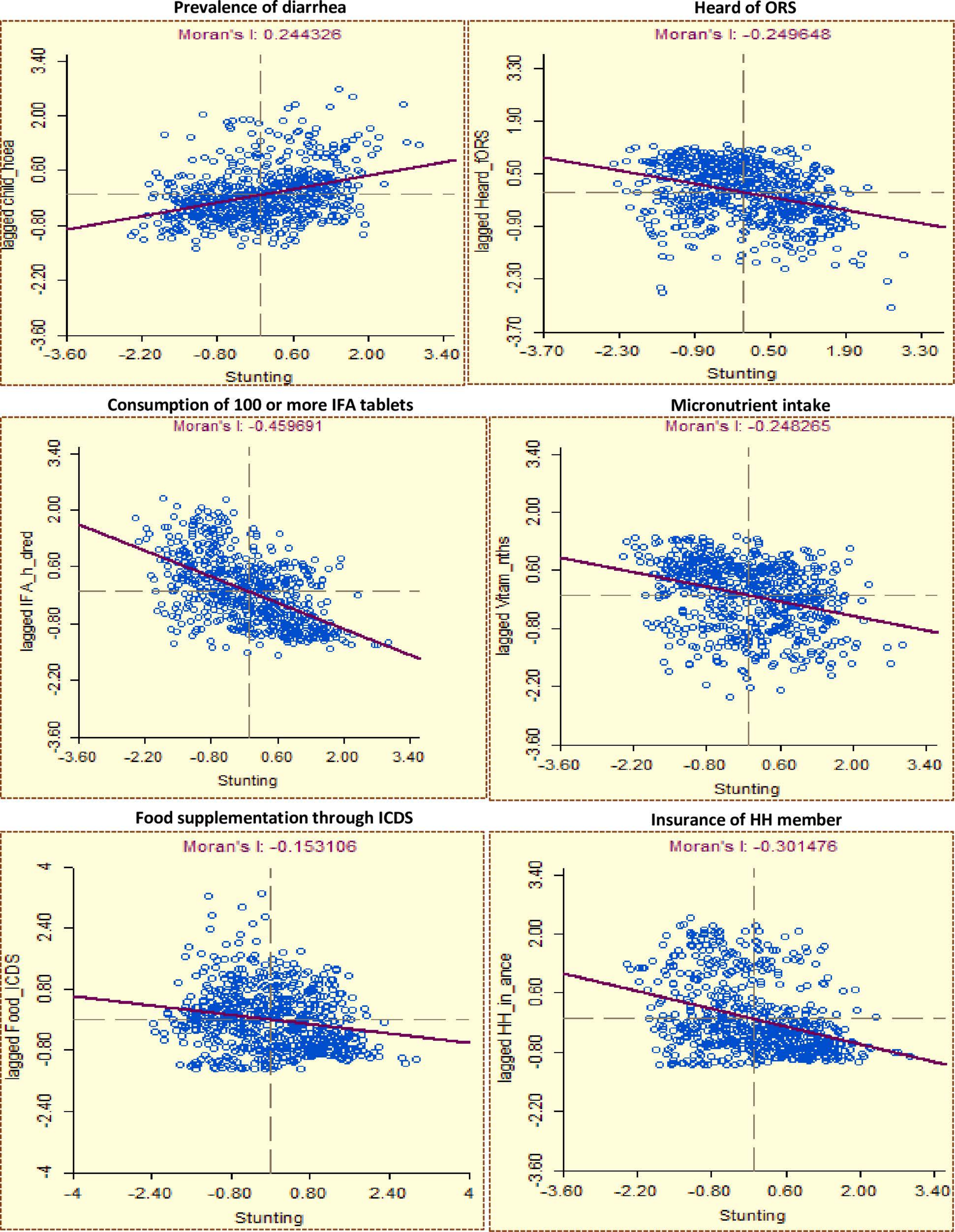

Prevalence of diarrhea
Moran's I: 0.244326
lagged child_hoea
Stunting
-3.60 -2.20 -0.80 0.60 2.00 3.40
Heard of ORS
Moran's I: -0.249648
lagged Heard_fORS
Stunting
-3.70 -2.30 -0.90 0.50 1.90 3.30
Consumption of 100 or more IFA tablets
Moran's I: -0.459691
lagged IFA_h_dred
Stunting
-3.60 -2.20 -0.80 0.60 2.00 3.40
Micronutrient intake
Moran's I: -0.248265
lagged Vitam_nths
Stunting
-3.60 -2.20 -0.80 0.60 2.00 3.40
Food supplementation through ICDS
Moran's I: -0.153106
lagged Food_ICDS
Stunting
-4 -2.40 -0.80 0.80 2.40 4
Insurance of HH member
Moran's I: -0.301476
lagged HH_in_ance
Stunting
-3.60 -2.20 -0.80 0.60 2.00 3.40

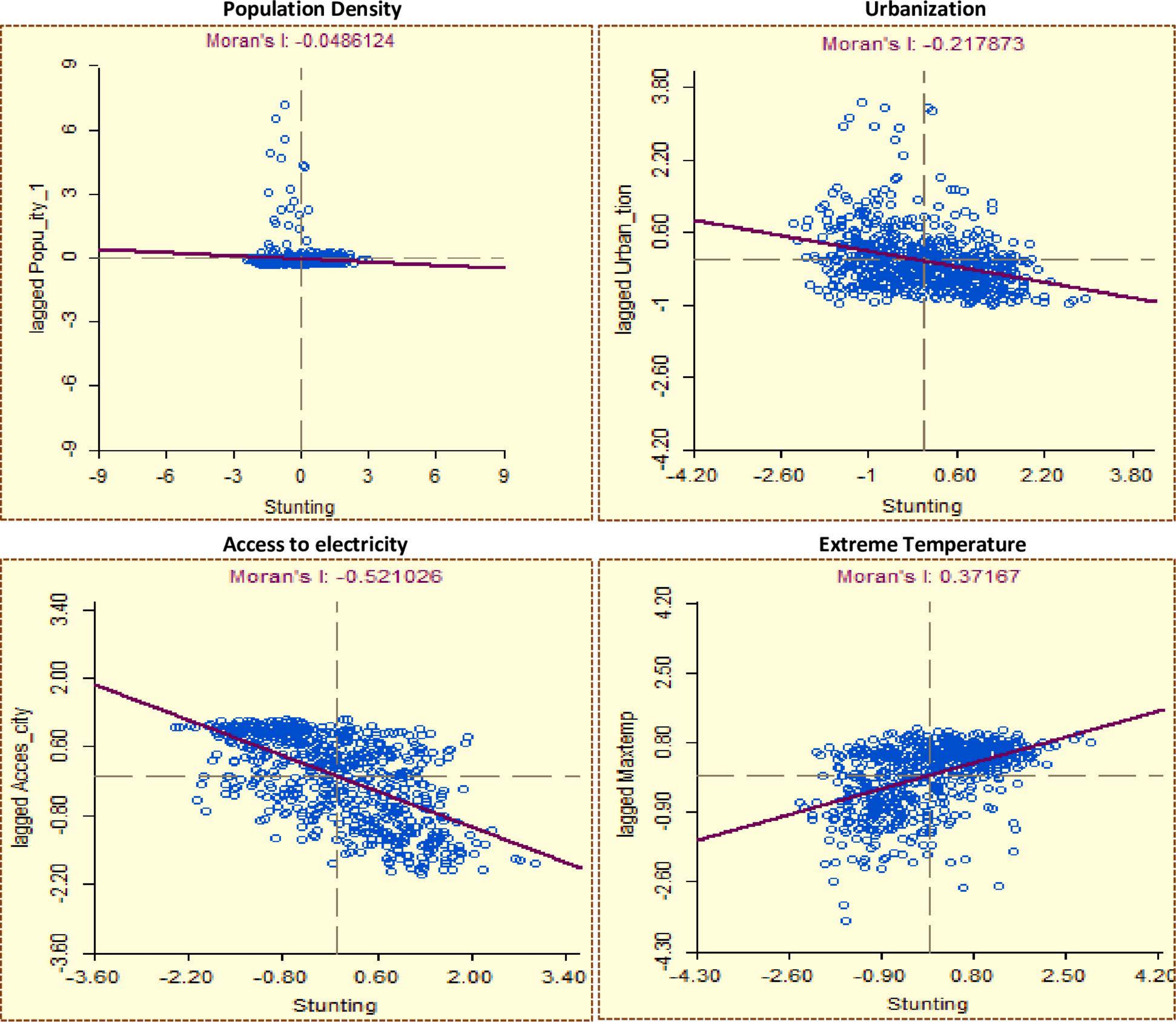

Population Density
Moran's I: -0.0486124
lagged Popu_ity_1
Stunting
Urbanization
Moran's I: -0.217873
lagged Urban_tion
Stunting
Access to electricity
Moran's I: -0.521026
lagged Acces_city
Stunting
Extreme Temperature
Moran's I: 0.37167
lagged Maxtemp
Stunting
